# Characteristics of COVID-19 comorbidities and severity profiles among pregnant women from a single-center cross-sectional study

**DOI:** 10.1097/MD.0000000000038636

**Published:** 2024-06-21

**Authors:** Amillia Siddiq, Vischila Geray D’lamanda, Muhamad Dwi Anggi, Lulu Eva Rakhmilla, Akhmad Yogi Pramatirta, Dini Pusianawati, Leni Lismayanti, Anggraini Widjajakusuma, Annisa Dewi Nugrahani, Dhanny Primantara Johari Santoso

**Affiliations:** a Department of Obstetrics and Gynecology, Faculty of Medicine, University of Padjadjaran – Dr. Hasan Sadikin General Hospital Bandung, West Java, Indonesia; b Faculty of Medicine, University of Padjadjaran – Dr. Hasan Sadikin General Hospital Bandung, West Java, Indonesia; c Department of Public Health (Epidemiology), Faculty of Medicine, University of Padjadjaran – Dr. Hasan Sadikin General Hospital Bandung, West Java, Indonesia; d Department of Clinical Pathology, Faculty of Medicine, University of Padjadjaran – Dr. Hasan Sadikin General Hospital Bandung, West Java, Indonesia; e Department of Internal Medicine, Faculty of Medicine, University of Padjadjaran – Dr. Hasan Sadikin General Hospital Bandung, West Java, Indonesia.

**Keywords:** comorbidities, COVID-19, CRP levels, maternal health, pregnancy

## Abstract

The study aimed to determine the characteristics of comorbidities, association between comorbidities and coronavirus disease 2019 (COVID-19), as well as characteristics of COVID-19 severity among pregnant women at a tertiary hospital in Bandung. We conducted a cross-sectional study by taking secondary data between January 2020 and December 2021 involving 278 pregnant women aged 16 to 45 years that confirmedly diagnosed with COVID-19 via RT-PCR. We collected information from the medical record on severity and comorbidities. The admission C-reactive protein (CRP) profiles were compared between the severe and nonsevere COVID-19 patients. This study employed bivariate analysis, *t* test, and multivariate analysis with logistic regression models. Of the 278 data included in this study, 120 cases had comorbidities. Most patients were asymptomatic (82%). Obesity was the most common comorbid proportion. Only hypertension as comorbid showed a significant association with symptomatic or asymptomatic COVID-19 (<0.05). Pregnant women with hypertension were 6 times more likely to show symptoms than those without hypertension (OR = 6.092; 95% CI 3.103–11.962). Pregnant women with comorbidities were at higher risk of cesarean sections and stillbirth. The CRP levels which were found to have statistically significant association with COVID-19 severity (<0.05). The domination of asymptomatic COVID-19 in pregnant women was found in this study. Hypertension comorbid has a significant association with COVID-19 symptoms. Maternal and neonatal outcomes appear to be influenced by maternal comorbidities. Moreover, the CRP levels were found to be significant risk factors for COVID-19 severity in pregnant women that might have association with comorbidities.

## 1. Introduction

Coronavirus disease 2019 (COVID-19) has become a pandemic responsible for increasing deaths worldwide for all populations including pregnant women. Over 762 million confirmed cases and more than 6.8 million fatalities had been reported globally as of April 2023.^[1, 2]^ The data from the Indonesian Society of Obstetrics and Gynecology or *Indonesian Society of Obstetrician and Gynecologist* (ISOG/POGI) show that from April 2020 to April 2021, there were 536 pregnant women with COVID-19. In January to July 2021, there were 205 pregnant women with COVID-19 in Tertiary Hospital in Bandung, with 4 cases resulting in death.^[2–5]^

People infected with COVID-19 may show variant clinical features ranging from no symptoms (asymptomatic), or symptomatic (mild to severe).^[6]^ The World Health Organization (WHO) classified COVID-19 based on the severity of cases into asymptomatic, mild, moderate, severe, and critical. This classification will influence the management directed at each specific populations based on their clinical status.^[7,8]^

In addition to adaptation for fetal growth and development, anatomical, physiological, and immunological changes in pregnancy can affect the risk and severity of certain infections, including respiratory pathogens infection like COVID-19 due to cardiopulmonary changes.^[9,10]^ Studies from the United States, National Data, and East Java in Indonesia showed that pregnant women with Severe Acute Respiratory Syndrome Coronavirus 2 (SARS-CoV-2) infection were most likely to be asymptomatic or only have mild symptoms.^[11–14]^

According to the WHO, pregnancy with comorbidities, including obesity, hypertension, diabetes mellitus, and risk factor advanced maternal age ≥ 35 years old, highlighted to increase the risk of developing severe illness of COVID-19 compared to those without. Obesity is associated with chronic inflammation and mechanical changes in lung function while hypertension and diabetes mellitus cause immune system dysfunction that increasing organ damage which results in increased severity and mortality in COVID-19 patients.^[15–17]^

Furthermore, infection of SARS-CoV-2 can cause systemic inflammation which significantly affects the patient’s immune response. C-reactive protein (CRP) is an acute phase protein which play roles as an early marker of inflammation or infection.^[18–20]^ CRP serum levels are routinely measured in the early diagnosis of pneumonia.^[21]^

Regarding to Centers for Disease Control and Prevention (CDC), pregnant women who are infected with COVID-19 are at higher risk of experiencing complications that can affect pregnancy and infant development.^[22]^ Several complications are significantly higher in pregnant women with COVID-19, including maternal death, preeclampsia, and premature birth. According to previous studies, a pro-inflammatory condition caused by SARS-CoV-2 infection may lead to preeclampsia and systemic endothelial dysfunction. Although SARS-CoV-2 infection may potentially result in exaggerated systemic inflammatory responses implicated in the pathophysiology of preterm delivery or an unfavorable environment for fetal growth and development. Some of these additional risks may be related to preeclampsia. Placental histopathologic results in individuals with COVID-19 at delivery have revealed placental fetal vascular malperfusion, which may be linked to preterm birth, stillbirth, and fetal development.^[23]^ In addition, pregnant women have an increased risk of serious illness from COVID-19 compared to nonpregnant women.^[24]^ Ahnach et al,^[25]^ in their research conducted at International University Cheikh Khalifa Hospital, CRP level was significantly related to the COVID-19 severity. Similarly, with the research conducted by Wang et al,^[26]^ CRP levels can reflect the disease severity and can be used as a key indicator for disease monitoring.

Moreover, there have been no previous studies adequately reported the characteristics of comorbidities and severity among pregnant women especially in Indonesia during pandemic due to restrictions on daily activities. Based on these issues, the authors aimed to conduct a study on the characteristics of comorbidities, association between comorbidities and COVID-19, as well as characteristics of COVID-19 severity among pregnant women at a tertiary hospital in Indonesia as a local data that could support global data.

## 2. Material and methods

A cross-sectional study was conducted in the largest public hospital of West Java, in Indonesia. All pregnant women admitted to the hospital with confirmed COVID-19 by RT-PCR cases from January 2020 to December 2021, were notified to our database. This study was approved by the local ethical ethics committee (No. 651/UN6.KEP/EC/2022 and No. 810/UN6.KEP/EC/2022). Informed consent was obtained from all the participants before the study began. The study exclusion criteria were incomplete medical record data and multifetal gestation. Data collected in this study were basic maternal data (maternal age, gestational age, and prepregnancy body weight), comorbidities, COVID-19 infection-related data (symptomatic or asymptomatic according to WHO classification and COVID-19 outcome which were survive or death), and pregnancy outcomes (delivery method; vaginal or cesarean section and neonatal outcome; term or preterm or stillbirth). For symptomatic group, we also did subgroup analysis regarding CRP levels between severe and nonsevere group.

### 2.1. Sampling methods

The sampling technique in this study was consecutive. Based on the formula, the minimum sample size in this study was 30 samples.^[27]^

### 2.2. Data collection and laboratory examination

The latex agglutination theory serves as the foundation for the CRP test. Human anti-CRP complexed with latex particles reacts visibly in 2 minutes when combined with a patient’s serum that contains CRP and measured the CRP quantitatively.

### 2.3. Data statistical analysis

For the descriptive analysis, we described variables as mean with standard deviation (SD), since the data were normally distributed and categorical variables as percentages and frequencies. The chi-square test was used to compare symptomatic and asymptomatic COVID-19 symptoms according to age, hypertension, obesity, and diabetes mellitus. This study employed bivariate analysis with *t* test and multivariate analysis with logistic regression models. Data analysis was performed using SPSS version 26.0 for Mac. A *P* < .05 was considered statistically significant in all analyses.

## 3. Results

A total of 309 pregnant women with confirmed COVID-19 met the inclusion and exclusion criteria of this study, of which 21 had incomplete medical record data and ten multifetal gestations. Two hundred seventy eight patients were included in this study (Table 1).

**Table 1:** Data characteristics of pregnant women with COVID-19 (n = 278).

Characteristics	Symptomatic (n = 50)	Asymptomatic (n = 228)	Total
Age, n (%)			
• <35 years	37 (17)	182 (83)	219
• ≥35 years	13 (22)	46 (78)	59
Gestational age, n (%)			
• Trimester 1	NA	6 (100)	6
• Trimester 2	1 (11)	8 (89)	9
• Trimester 3	49 (19)	214 (81)	263

**Figure 1.**
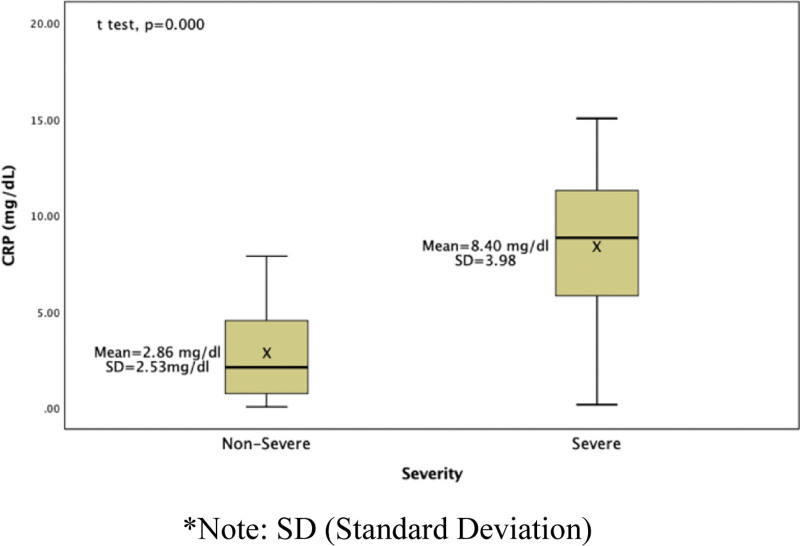
Difference of CRP level in nonsevere and severe group in pregnant women with COVID-19. COVID-19 = coronavirus disease 2019, CRP = C-reactive protein.

In symptomatic subgroup analysis, this study was consisted of 35 severe patients and 15 nonsevere patients. The proportion of pregnant women with nonsevere group COVID-19 infection was higher than those severe group, 70.1% versus 29.9%. The distributions of CRP levels among patient in different severity are presented in Figure 1. The levels of CRP on admission were significantly different between the 2 groups. The results of statistical analysis using the independent *t* test between CRP and COVID-19 severity in pregnant women showed significant difference between CRP and COVID-19 severity in pregnant women (<0.001). The level of CRP in the severe group (Mean 8.40; SD 3.98 mg/dL; 95% CI 2.215–3.510) was 3 times higher than in the nonsevere group (Mean 2.86; SD 2.53 mg/dL; 95% CI 6.788–10.002). Data distribution between group was normal.

### 3.1. Analysis of comorbidities of COVID-19 among pregnant women

The proportion of pregnant women with asymptomatic COVID-19 infection was higher than those in symptomatic groups, 82% versus 18%. Of 50 pregnant women had symptoms, 9 were in severe cases.

The total number of pregnant women with comorbidities is 120, of which 33 (28%) have more than 1 comorbid. Pregnant women without comorbidities had a higher proportion compared to those with comorbidities, 57% versus 43%. The most common comorbidity was obesity (71%). Data collected from medical records were body height and weight prepregnancy (BMI > 30) and obesity diagnosis in pregnancy. The proportion of comorbid hypertension in both symptomatic and asymptomatic groups was close to 50%. Hypertension comorbid included 19 chronic hypertension superimposed preeclampsia, 13 chronic hypertension, 12 preeclampsia, 6 eclampsia, 3 HELLP syndrome, and 1 gestational hypertension (Table 2).

**Table 2:** Comorbidities of pregnant women with COVID-19.

	Symptomatic n (%)	Asymptomatic n (%)	Total
Obesity	21 (25)	64 (75)	85
Hypertension	24 (44)	30 (56)	54
HIV/AIDS^a^	NA	4 (100)	4
Diabetes mellitus	NA	2 (100)	2
Chronic heart disease	1 (50)	1 (50)	2
Hepatitis B	1 (50)	1 (50)	2
Chronic pulmonary disease	NA	1 (100)	1
Systemic lupus erythematosus	NA	1 (100)	1
Nontoxic goiter	NA	1 (100)	1
Thalassemia	NA	1 (100)	1

a HIV/AIDS = human immunodeficiency virus/acquired immunodeficiency syndrome

There was no difference data proportion between symptomatic and asymptomatic groups. The results of statistical analysis using the Chi-Square test between risk factor advanced maternal age and COVID-19 symptoms showed no significant association between risk factor advanced maternal age and symptomatic or asymptomatic COVID-19 (>0.05). Advanced maternal age women were 1.4 times more likely to show symptoms than the <35 years group (OR 1.39; 95% CI 0.684–2.827).

However, hypertension comorbid showed a significant association with symptomatic or asymptomatic COVID-19, which meant pregnant women with hypertension were more likely to show symptoms and those without showed the opposite (<0.05). Pregnant women with hypertension were 6 times more likely to show symptoms than those without hypertension (OR 6.092; 95% CI 3.103–11.962).

Although obesity was the most common comorbid, the statistical analysis showed that obesity had no significant association with symptomatic or asymptomatic COVID-19 (> 0.05). Pregnant women with obesity in this study were 1.8 times more likely to show symptoms than those without obesity (OR 1.856; 95% CI 0.987–3.489) (Table 3).

**Table 3:** Association between risk factor, comorbidities, and symptoms in pregnant women with COVID-19 (bivariate).

Variable	Symptomatic n (%)	Asymptomatic n (%)	OR	95% CI	*P*
Age ≥ 35 yr	13 (22)	46 (78)	1.39	0.684 to 2.827	.362
Hypertension	24 (44)	30 (56)	6.092	3.103 to 11.962	.0001
Obesity	21 (25)	64 (75)	1.856	0.987 to 3.489	.053
Diabetes mellitus	NA	2 (100)	NA	NA	1

CI = confidence interval, OR = odds ratio.

**Figure 2.**
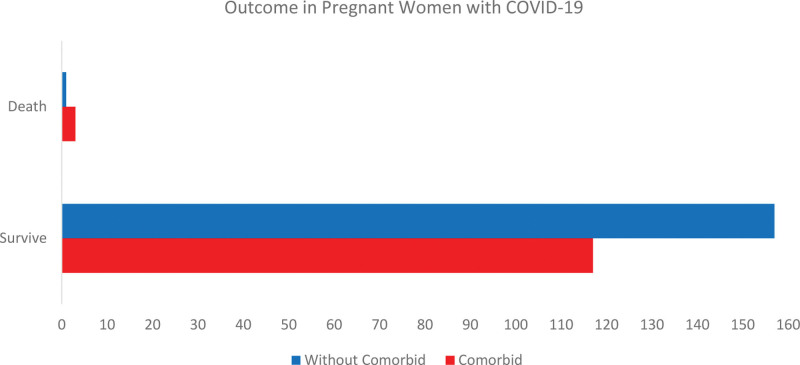
Outcome in pregnant women with COVID-19. COVID-19 = coronavirus disease 2019.

Of the 274 pregnant women who survived, 117 (43%) had comorbidities and 157 (57%) had no comorbidities. The majority of maternal deaths were from pregnant women with comorbidities group (75%) as shown in Table 4. All the maternal deaths were due to respiratory failure that leading to cardiac arrest which related to COVID-19 severe symptoms. In general, pregnant women with comorbidities were 1.7 times more likely to have a worse prognosis than the group without (Odds ratio 1.756; 95% CI 0.981–3.144) (Fig. 2).

**Table 4:** Association between risk factor, comorbidities, and symptoms in pregnant women with COVID-19 (multivariate) analysis.

Variable	Bivariate analysis			Multivariate analysis	
	Crude OR	95% CI	*P*	Adj OR	95% CI
Age ≥ 35	1.39	0.684 to 2.827	.362	NA	NA
Hypertension	6.092	3.103 to 11.962	.0001	6.092	3.103 to 11.962
Obesity	1.856	0.987 to 3.489	.053	NA	NA

CI = confidence interval, OR = odds ratio.

A multivariate logistic regression analysis showed hypertension was comorbid that had a reasonably significant association with COVID-19 symptoms.

Apart from obstetrical indications, pregnant women with comorbidities were more likely to give birth by cesarean section than vaginal delivery, in the group without showed the opposite proportion. No difference percentages were observed in neonatal outcomes among comorbidities and without comorbidities. Preterm neonatal outcomes from both groups showed relatively same percentage, 26% versus 28%, respectively. Stillbirth was dominated by pregnant women with comorbidities (67%) (Table 5).

**Table 5:** Maternal and neonatal outcomes.

	Comorbid (n = 115)	Without comorbid (n = 137)
Delivery method, n (%)		
• Vaginal	43 (36)	78 (64)
• C-Section^a^	72 (55)	59 (45)
Neonatal outcome, n (%)		
• Aterm	81 (46)	97 (54)
• Preterm	30 (44)	38 (56)
• Stillbirth	4 (67)	2 (33)

a C-Section: caesarean section.

## 4. Discussions

In this study, interestingly, only 18% of pregnant women with COVID-19 had signs and symptoms. The domination of asymptomatic COVID-19 in pregnant women found in this study was consistent with a study from New York, which found that 87.9% were asymptomatic.^[12]^ This is in line with the national data from the Indonesian Society of Obstetrics and Gynecology (POGI) reported 51.9%, and a cohort retrospective study from a major referral hospital in East Java reported that 75.8% of pregnant women with COVID-19 were asymptomatic.^[13,14]^ Anatomical adaptations in the cardiopulmonary system during pregnancy increase susceptibility to respiratory infections. The increase in estrogen and progesterone level caused respiratory tract edema. In addition, uterine expansion superiorly suppressed the diaphragm resulting in the limitation of lung expansions.^[28–30]^ The following factors may cause asymptomatic SARS-CoV-2 infections in pregnancy.

It was demonstrated in this study that in patients diagnosed with COVID-19, CRP elevation upon admission was common and was associated with increased disease severity. CRP is a pentameric protein synthesized by the liver, whose level rises in response to inflammation. There are other pathological or nonpathological processes that increases the CRP level, such as in patients with infections, malignancies, inflammations, and rheumatologic disorders. Previous studies have shown that pregnancy can also cause CRP levels to increase. CRP value in normal pregnancy have a median of 0.7–0.9 mg/dL. Whereas, the median CRP value for women in labor at term was 1.3 mg/dL.^[31]^ The reason why such as physiological changes occur during labor remains unclear, but elevated estrogen and prostaglandin serum concentration could stimulate CRP synthesis in the liver which could explain those findings.^[32]^

According to the comparison based on the severity of the disease, effect of several reported indicators for disease severity were detected and CRP level had a significant difference in severe patients. Recent studies have reported that cases of severe COVID-19 exhibit increased plasma levels of interleukin (IL) 2, IL-6, IL-7, IL-10, granulocyte colony-stimulating factor (GCSF), tumor necrosis factor ɑ (TNF-ɑ), and others.^[32,33]^ Thus, elevated CRP at admission may both reflect significant inflammation and itself drive further inflammation. Findings from this study were consistent with recent publications, which indicated that the CRP level on admission have significant difference between COVID-19 severity.^[32,33]^ The difference and one of this study’s novelties is that this study also includes pregnant women subject. We recommend that the levels of CRP should be measured, as they can help diagnose the COVID-19 severity in pregnant patients

Obesity is associated with reduced expiratory reserve volume, inhibition of diaphragm excursion, ventilation limitation, triggered inflammatory cytokines, and oxidative stress. In addition, it is associated with other comorbidities such as hypertension, diabetes mellitus, and dyslipidemia resulting in an increased risk of severe COVID-19 symptoms.^[25]^ In this study, pregnant women with obesity were 1.8 times more likely to show symptoms than those without obesity. Obesity in pregnancy closely related with adverse pregnancy outcome such as macrosomic infants.^[26]^

A retrospective cohort study involving 17 hospitals in the United States reported that hypertension comorbid tended to be higher in pregnant women with COVID-19 than in the non-COVID-19 group, 6.9% versus 5.5%.^[24]^ The entry of SARS-CoV-2 into cells is a complicated process that involves the binding of glycoprotein S viral to the ACE2 receptor host. The pathophysiology of hypertension consists of the activation of ACE2 that causes an increase in the activity of the renin–angiotensin–aldosterone system (RAAS), resulting in vasoconstriction, which causes organ damage. In addition, releasing immune cells such as monocytes, macrophages, CD8+ T cells, and CD4+ T cells results in vascular dysfunction and organ damage.^[19]^

In addition, SARS-CoV-2 infection in pregnancy causes RAAS dysregulation that associated with adverse pregnancy outcomes. In this study, pregnant women with hypertension were 6 times more likely to show symptoms than those without hypertension. This is consistent with the findings from a study that hypertension can increase organ damage and immune system dysfunction in COVID-19 patients.^[34,35]^

Pregnant women with comorbidities had a worse prognosis than those without comorbidities, this study is consistent with a multinational retrospective cohort study of 887 samples involving 25 countries in Europe, America, Asia, and Australia, pregnant women with advanced maternal age and comorbid diabetes mellitus, hypertension, and autoimmune diseases associated with adverse maternal outcomes than in low-risk pregnancy (OR 1.52; 95% CI 1.03–2.24; *P* = .035). In another multinational cohort study involving 18 countries from 2130 samples, pregnant women with COVID-19 were 22 times more likely to die than non-COVID-19 group (RR 22.3: 95% CI 2.99–172).^[36,37]^

COVID-19 management in pregnancy based on severity is carried out in a multidisciplinary manner along with the hospital’s COVID-19 task force team. Pregnancy termination is determined based on obstetric indications and maternal therapies include supplementation, antivirals, antibiotics, anti-inflammatories, anti-interleukin 6 receptors, anticoagulants, to convalescent plasma therapy and immunoglobulin in more severe cases. However, COVID-19 is not an indication for cesarean section. The mode of delivery should be considered based on obstetric indications, comorbidities, and the severity of the disease. In pregnant women with COVID-19, a cesarean section is most commonly indicated due to maternal hypoxemia. In this study, pregnant women with comorbidities tended to give birth by cesarean section than vaginal delivery, however, the group without comorbidities showed the opposite proportion. This is in line with a study involving 926 pregnant women with COVID-19; the high-risk group was at higher risk of cesarean section, 70.7%, nevertheless the low-risk group was at higher risk of vaginal delivery method, 68.1%. High-risk group included maternal age ≥ 35 years, obesity, hypertension, diabetes mellitus, lung disease, kidney disease, cardiovascular disease, and immunological disorders.^[38]^

Preterm delivery < 37 weeks in this study tended to be higher than in the general population in Indonesia before the pandemic. The increase in the risk of preterm delivery is associated with the inflammatory response to COVID-19 that triggered fetal distress. A cohort retrospective study from a major referral hospital in East Java reported that preterm delivery tended to be higher in the COVID-19 group than in the non-COVID-19 group, although the difference was not statistically significant.^[39]^

Stillbirth was dominated by pregnant women with comorbidities; this study is in line with the CDC reporting that among 1.249.634 pregnant women in the United States, women with COVID-19 are 1.9 times more at risk of experiencing a stillbirth than those without COVID-19. SARS-CoV-2 is associated with hypoperfusion and inflammation of the placenta which may be related to mechanisms of stillbirth in pregnant women with COVID-19.^[40]^

Severe COVID-19 in pregnancy is relatively rare, but it has a significant negative impact on both the mother and fetus. In line with Vousden et al^[41]^, this study identifies several characteristics that increase the risk of severe COVID-19 in pregnancy and demonstrates promising vaccine efficacy. In keeping with the wider literature, most women admitted to the hospital with COVID-19 are in their third trimester, and they should be informed of the greater risk at this time to allow consideration of earlier vaccination and measures to reduce exposure wherever possible.

The principal comorbidities among pregnant women in the study were obesity and hypertension. Hypertension comorbid has a significant association with COVID-19 symptoms, therefore, future studies are needed to explore about hypertension and COVID-19 especially in pregnant women. Maternal and neonatal outcomes appear to be influenced by maternal comorbidities. There may be some possible limitations in this study. First, data were collected in 2020 to 2021 prior to wide circulation of the B.1.617.2 (Delta) variant of SARS-CoV-2, which may have different effects from the latest variant.

In general, this study had a sufficient sample size; however, the sample was not sufficient enough to analyze diabetes mellitus comorbid. Another limitation of this study was only focused on symptomatic and asymptomatic COVID-19, and this study only did subgroup analysis in symptomatic group (CRP levels between severe and nonsevere group). Our study did not include postpartum women referred to our hospital, and given that the postpartum period may have a greater risk of poor outcomes, future studies are recommended in this area. Moreover, this study should have a further investigation since in normal pregnancies there is also an increased in CRP levels.

## 5. Conclusion

The domination of asymptomatic COVID-19 in pregnant women was found in this study. Hypertension comorbid has a significant association with COVID-19 symptoms. Maternal and neonatal outcomes appear to be influenced by maternal comorbidities. Moreover, the CRP levels were found to be significant risk factors for COVID-19 severity in pregnant women that might have association with comorbidities. We recommend that the levels of CRP should be measured in combination with disease’s comorbidities to diagnose and predict COVID-19 outcome among pregnant women.

## Author contributions

**Conceptualization:** Amillia Siddiq, Vischila Geray D’lamanda, Muhamad Dwi Anggi, Akhmad Yogi Pramatirta, Dini Pusianawati, Leni Lismayanti, Anggraini Widjajakusuma, Dhanny Primantara Johari Santoso.

**Data curation:** Amillia Siddiq, Vischila Geray D’lamanda, Muhamad Dwi Anggi, Lulu Eva Rakhmilla, Akhmad Yogi Pramatirta, Dini Pusianawati, Leni Lismayanti, Anggraini Widjajakusuma, Annisa Dewi Nugrahani.

**Formal analysis:** Amillia Siddiq, Vischila Geray D’lamanda, Muhamad Dwi Anggi, Lulu Eva Rakhmilla, Akhmad Yogi Pramatirta, Dini Pusianawati, Leni Lismayanti, Anggraini Widjajakusuma.

**Funding acquisition:** Amillia Siddiq, Vischila Geray D’lamanda, Muhamad Dwi Anggi.

**Investigation:** Amillia Siddiq, Vischila Geray D’lamanda, Muhamad Dwi Anggi, Akhmad Yogi Pramatirta, Dini Pusianawati, Leni Lismayanti, Anggraini Widjajakusuma.

**Methodology:** Amillia Siddiq, Vischila Geray D’lamanda, Muhamad Dwi Anggi, Lulu Eva Rakhmilla, Akhmad Yogi Pramatirta, Dini Pusianawati, Leni Lismayanti, Anggraini Widjajakusuma.

**Project administration:** Amillia Siddiq, Vischila Geray D’lamanda, Muhamad Dwi Anggi, Dini Pusianawati.

**Resources:** Amillia Siddiq, Vischila Geray D’lamanda, Muhamad Dwi Anggi.

**Supervision:** Amillia Siddiq, Lulu Eva Rakhmilla, Akhmad Yogi Pramatirta, Dini Pusianawati, Leni Lismayanti, Anggraini Widjajakusuma, Dhanny Primantara Johari Santoso.

**Validation:** Amillia Siddiq, Lulu Eva Rakhmilla, Akhmad Yogi Pramatirta, Dini Pusianawati, Leni Lismayanti, Anggraini Widjajakusuma.

**Writing – original draft:** Amillia Siddiq, Vischila Geray D’lamanda, Muhamad Dwi Anggi, Akhmad Yogi Pramatirta, Dini Pusianawati, Leni Lismayanti, Anggraini Widjajakusuma, Annisa Dewi Nugrahani, Dhanny Primantara Johari Santoso.

**Writing – review & editing:** Amillia Siddiq, Vischila Geray D’lamanda, Muhamad Dwi Anggi, Annisa Dewi Nugrahani.

**Software:** Vischila Geray D’lamanda, Muhamad Dwi Anggi, Lulu Eva Rakhmilla, Annisa Dewi Nugrahani.

**Visualization:** Vischila Geray D’lamanda, Muhamad Dwi Anggi.
